# The *Lactobacillus plantarum* P-8 Probiotic Microcapsule Prevents DSS-Induced Colitis through Improving Intestinal Integrity and Reducing Colonic Inflammation in Mice

**DOI:** 10.3390/nu16071055

**Published:** 2024-04-04

**Authors:** Han Wang, Yaxuan Sun, Xuan Ma, Tianyu Yang, Feng Wang

**Affiliations:** Department of Food Science, School of Biochemical Engineering, Beijing Union University, Beijing100023, China; wanghan2075@163.com (H.W.); sunxx@buu.edu.cn (Y.S.); shtmaxuan@buu.edu.cn (X.M.); yangtianyu@buu.edu.cn (T.Y.)

**Keywords:** probiotic microcapsules, colitis, intestinal mucosal barriers, inflammation, NLRP3

## Abstract

Probiotics, recognized as beneficial and active microorganisms, often face challenges in maintaining their functionality under harsh conditions such as exposure to stomach acid and bile salts. In this investigation, we developed probiotic microcapsules and assessed their protective effects and underlying mechanisms in a murine model of dextran sulfate sodium (DSS)-induced colitis using male C57BL/6J mice. The administration of the probiotic microcapsules significantly mitigated body weight loss, prevented colon length shortening, decreased the disease activity index scores, and reduced histopathological scores in mice with DSS-induced colitis. Concurrently, the microencapsulated probiotics preserved intestinal barrier integrity by upregulating the expressions of tight junction proteins ZO-1 and occludin, as well as the mucus layer component MUC-2. Moreover, the treatment with probiotic microcapsules suppressed the activation of the NLRP3 inflammasome signaling pathway in the context of DSS-induced colitis. In conclusion, these findings support the utilization of probiotic microcapsules as a potential functional food ingredient to maintain the permeability of the intestinal barrier and alleviate colonic inflammation in UC.

## 1. Introduction

Inflammatory bowel diseases (IBDs) are defined by chronic inflammation of the gastrointestinal mucosa [1]. These inflammatory bowel diseases primarily consist of two main types: ulcerative colitis (UC) and Crohn’s Disease [2]. UC is conceptualized as an intricate outcome arising from the multifactor influences, notably environmental triggers, genetic disorder, gut microbiota, and immune system response deficiency. Among these contributors, the chronic inflammation of the intestinal mucosa and intestinal immune system dysfunctions, reduction of mature goblet cells, and defects in mucus secretion contribute to the development and progression of UC [3,4,5].

Ulcerative colitis (UC) is characterized by disrupted epithelial integrity and a decreased number of tight junction (TJ) strands. These apical tight junctions play a critical role in the selective permeability of the gastrointestinal barrier for restricting the passage of microorganisms, inhibiting the diffusion of toxins, and controlling the absorbance and penetration of digested nutrients [6]. TJs are assembled through a complex interaction involving transmembrane proteins, such as occludins, which interact closely with auxiliary proteins known as zonula occludens (ZOs) [7,8,9,10]. These proteins collectively contribute to maintaining the integrity of the intestinal barrier and regulating intestinal permeability. Consequently, bolstering the intestinal mucosal barrier function could potentially represent a critical therapeutic approach for treating UC [11].

Many researchers have demonstrated that the disruption of the intestinal immune response and the inflammation triggered by dysbiosis are also significant contributing factors to the impairment of the intestinal mucosal epithelial barrier and the advancement of IBD [12,13]. Among them, a critical characteristic in the pathogenesis of colitis is the heightened presence of inflammatory cytokines [14]. The NOD-like receptor family pyrin domain-containing 3 (NLRP3) inflammasome signaling pathway plays a central role in the onset and progression of intestinal inflammation [15]. The NLRP3 inflammasome complex in the cytosol consists of the innate immune receptor protein NLRP3, the adaptor protein ASC, and the inflammatory protease caspase-1, which is activated in response to a variety of triggers, including microbial infection, endogenous danger signals, and environmental stimuli [16]. Consequently, regulating the production of inflammatory cytokines by targeting and modulating different inflammatory signaling pathways presents a promising therapeutic approach for the management and treatment of colitis.

Clinically administered treatments, including corticosteroids, 5-aminosalicylic acid (5-ASA) derivatives, and immunosuppressive medications, have proven effective in managing UC symptoms. However, the chronic use of such medications is commonly related to a wide range of adverse effects, such as allergic reactions, fever, hypertension, and liver complications. These symptoms prompt scientists to explore alternative or complementary therapies that are effective, safe, and have fewer long-term side effects [17,18,19,20].

In this regard, several studies have demonstrated the therapeutic effects of various probiotic bacterial strains on ulcerative colitis. Probiotic *Lactobacillus plantarum* P-8 strain has been documented to exhibit several potential therapeutic effects as anti-inflammatory, antioxidant, and improve the balance and diversity of gut microbiota. Furthermore, probiotic bacteria promote the response of the mucosal immune system by producing protective immunoglobulins (Ig) and bacteriocins in the intestinal mucous membrane, which leads to preventing the proliferation of harmful bacteria from reaching the lamina propria through competitive inhibition. These properties make *Lactobacillus plantarum* P-8 a promising remedy for the treatment of certain gastrointestinal disorders [21,22,23]. The main challenge in using *Lactobacillus plantarum* P-8 for these purposes is their sensitivity to low pH and high concentration of bile salts in the gastrointestinal tract. To overcome such problems, a microencapsulation technique has been applied to protect probiotics during passage through the gut and colon, thereby ensuring their safe delivery to the intended target [24].

In this study, we aimed to encapsulate probiotic L. plantarum P-8 bacteria using gelatin and gum Arabic as encapsulating materials. We then studied the effect of orally administering these probiotic microcapsules on improving intestinal integrity and reducing colonic inflammation in mice, and also clarified the underlying mechanism of action.

## 2. Materials and Methods

### 2.1. Microcapsule Preparation Method

#### 2.1.1. Preparation of Bacterial Suspension of *L. plantarum* P-8

The cryopreserved bacterial strains were activated by sterilized MRS broth and incubated at 37 °C for 24 h. Then, the culture was inoculated into a freshly sterilized MRS broth media at a 5% inoculum ratio and incubated at 37 °C for 16 h until it reached the logarithmic growth phase. To collect the bacteria, the cultivated culture was centrifuged at 8000 rpm, at 4 °C for 10 min. The supernatant was discarded, and the harvested bacteria were resuspended in sterile normal saline to create a bacterial suspension.

#### 2.1.2. Encapsulation of *L. plantarum* P-8

The preparation of microcapsules was performed as described by Zhang Min [25], with slight modifications. Briefly, a 2% (*w*/*v*) gelatin (GE) solution was prepared and stirred with heating at 40 °C for one hour. Subsequently, after adding the suspension of P-8 gradually into the GE solution, 2% (*w*/*v*) gum Arabic (GA) was blended with GE at a 1:2 ratio of biopolymers. To enhance the electrostatic interaction between GE and GA, the pH of the combined solution was adjusted to 4.3 by 10% (*w*/*v*) acetic acid. The resultant microcapsule suspension was lyophilized for 24 h to produce the microcapsules.

### 2.2. Physical and Chemical Properties of Microcapsules

#### 2.2.1. Survival Rate after Microencapsulation

One gram of freeze-dried powder was suspended in 9 mL of sterile saline solution to release P-8. The diluted suspension was inoculated onto MRS agar and incubated at 37 °C for 48 h. The survival rate (log CFU/g) was calculated according to the following equation [26]:

Survival rate = N/N0 × 100%(1)

where N is the encapsulated viable bacteria count after encapsulation (log CFU/g) and N0 is the initial viable bacteria count before encapsulation (log CFU/g).

#### 2.2.2. Characterization of Microcapsules by Scanning Electron Microscopy (SEM)

The lyophilized *Lactobacillus plantarum* P-8, bare capsules, and P-8 microcapsules were each affixed onto the specimen holder using conductive adhesive. Under vacuum conditions, these samples underwent vertical gold sputtering. Subsequently, their microscopic morphology was analyzed using a SEM (S-570, HITACH Company, Tokyo, Japan), operated at an acceleration voltage of 10 kV to provide detailed imaging.

#### 2.2.3. Survival Rate of Free Probiotic and Probiotic Microcapsules in Simulated Gastrointestinal Conditions

The survival rates of *Lactobacillus plantarum* P-8 and P-8 microcapsules in simulated gastrointestinal tract were tested according to R. Rajam [27]. Preparation of simulated gastric juices included NaCl (0.2%, *w*/*v*), Pepsin (0.3%, *w*/*v*), and adjusting the pH of the solution to 2.0 with 1 M HCl. Preparation of simulated intestinal juices included NaCl (0.2%, *w*/*v*), trypsin (0.2%, *w*/*v*), bile salt (0.3%, *w*/*v*), and adjusting the pH of the solution to 8.0 with 1 M NaOH. One gram of both *Lactobacillus plantarum* P-8 and P-8 microcapsules were incubated in 9 mL of simulated gastric and intestinal juices at 37 °C and vigorously shaken at 100 rpm. Then, 1 mL was taken at 0, 1, 2, and 3 h to evaluate the total viable bacterial count.

#### 2.2.4. Survival Rate of Free Probiotic and Probiotic Microcapsules in High Temperature Conditions

One gram of *Lactobacillus plantarum* P-8 and P-8 microcapsules were incubated at 55 °C and 65 °C, respectively, for 30 min to evaluate the total viable bacterial count.

### 2.3. Animal Experimental Design and DSS Induced Colitis Model in Mice

Male C57BL/6J mice (20 ± 2 g of average body weight; aged at 7 weeks) purchased from Beijing Vital River Laboratory Animal Technology Co., Ltd (Beijing, China). were kept in a 12 h light cycle with food and water ad libitum. Animal feed was purchased from Beijing HFK Bio-Technology Co., Ltd (Beijing, China). According to the standard animal feeding requirements, one mouse per cage was kept aseptically. After 1 week of adaptive feeding, 40 mice were randomly divided into five groups: control (Con), DSS-induced colitis model (DSS), empty capsule treatment for DSS-induced mice (EC), *Lactobacillus plantarum* P-8 probiotic treatment for DSS-induced mice (P), and probiotic microcapsules treatment for DSS-induced mice (PM) The DSS, EC, P, PM groups were administered with 300 μL of physiological normal saline, 0.3 g/mL empty capsule solution, 3 × 10^7^ CFU live *Lactobacillus plantarum* P-8, and P-8 microcapsules with the same number of viable bacteria count for 21 days. In addition, the mouse colitis model was free to drink 2.5% (*w*/*v*) DSS (MW: 36,000–50,000 Da, MP Biomedicals, California, UK) solution from day 14 to 21. The protocol was approved by the Functional Testing Center of Health Food, College of Applied Arts and Sciences, Beijing Union University (JCZX11-2309-5, 12 September 2023).

Throughout the DSS oral administration, the body weight, food intake, and disease activity index (DAI) of the mice were consistently monitored and recorded. Upon completion of the experimental period, the mice were euthanized humanely via cervical dislocation. And, the colonic segments were carefully excised and stored at a temperature of −80 °C for further experimentation.

#### 2.3.1. Disease Activity Index (DAI)

During the DSS treatment, the body weight, stool consistency, and presence of fecal occult blood were monitored. An unbiased observer assessed the disease activity index (DAI) score based on Table 1. Additionally, the colon length of the mice was accurately measured and visually documented through photography.

#### 2.3.2. Histopathology Analysis

The distal segment of the colon was extracted from each mouse and immediately immersed in a tissue fixation solution (Servicebio, Wuhan, China, G1101-15ML). After embedding in paraffin, the distal segments were sectioned into 4-micrometer-thick slices. The paraffin-embedded colon sections were then stained using the Hematoxylin–Eosin (HE) staining protocol as previously described [28] and the slides were assessed for histological damage according to a standardized scoring system. The histopathology score system utilized in this study is detailed in Table 2.

#### 2.3.3. Immunofluorescence

After removal of the wax and rehydration, colon sections were immersed in a citric acid buffer solution and then subjected to microwave antigen retrieval. These sections were then processed for immunostaining using an anti-MUC-2 antibody (Servicebio, Wuhan, China, GB11344). Subsequently, the treated sections were rinsed with phosphate-buffered saline (PBS) and incubated with Cy3-labeled goat anti-rabbit IgG secondary antibody. To stain the cell nuclei, the sections were counterstained with 4′,6-diamidino-2-phenylindole (DAPI). Fluorescence images of the stained tissues were captured using a standing fluorescence microscope (Nikon, Tokyo, Japan, NIKON ECLIPSE C1) and scanner (3DHISTECH, Budapest, Hungary, Pannoramic MIDI). The analysis was performed using ImageJ software (version win-64).

#### 2.3.4. Immunohistochemistry

The dewaxing and rehydrating colon sections were immersed in a citric acid solution and subjected to microwave to perform heat-induced epitope retrieval. Following this, the sections were incubated with primary antibodies against ZO-1 (Servicebio, Wuhan, Hubei, #GB111402) and anti-occludin (proteintech, Wuhan, China, #27260-1-AP). The sections were then rinsed with water, dehydrated through graded alcohols and cleared in xylene. Finally, the glass slides were mounted with neutral resin (Servicebio, Wuhan, China, G1404) and examined under a microscope.

#### 2.3.5. Enzyme-Linked Immunosorbent Assay (ELISA)

Blood samples were drawn from the orbital sinuses of multiple mice and collected into centrifuge tubes. The samples were then centrifuged at 2000 rpm for 10 min each. Following centrifugation, the separated sera were used for the detection of cytokine biomarkers including Interleukin-1β (IL-1β), Interleukin-2 (IL-2), and Tumor Necrosis Factor-alpha (TNF-α) using ELISA test kits (Cusabio, Wuhan, China).

#### 2.3.6. Western Blot

An aliquot of 50 mg of colon tissue was mixed with pre-cooled RIPA lysate buffer containing protease inhibitors (bioshark, Beijing, China) and homogenized using a high speed and low temperature tissue grinder (Servicebio, Wuhan, China). The mixture was kept at 4 °C for 0.5 h. Then, the samples were centrifuged at 12,000 rpm under cooling conditions (4 °C) for 15 min. The protein content in the supernatant was quantified using the BCA method. The mixture was adjusted to 40 µg/10μL for the following step.

The proteins were fractionated using a dual gradient SDS-polyacrylamide gel electrophoresis (8% and 10%) for approximately 2 h. Upon completion, the protein fractions were electrophoretically transferred onto a polyvinylidene difluoride (PVDF) membrane. The transferred PVDF membrane was blocked by immersion in a 5% bovine serum albumin (BSA) solution for 2 h at ambient temperature to minimize non-specific binding. Thereafter, the membrane was incubated with primary antibodies tailored to target specific proteins, namely ZO-1 (proteintech, Wuhan, China, 21773-1-AP), occludin (proteintech, Wuhan, China, 27260-1-AP), NLRP3 (Cell Signaling Technology, Massachusetts, UK, 15101), caspase-1 (Cell Signaling Technology, Massachusetts, UK, 24232), ASC (Cell Signaling Technology, Massachusetts, UK, 67824), and β-actin (Servicebio, Wuhan, China, P60710) at 4 °C for 14 h. Following this, the PVDF membrane was incubated with secondary antibody for 2 h at ambient temperature (proteintech, Wuhan, China, SA00001-2).

### 2.4. Statistical Analysis

The outcomes of all experiments are presented as the mean values along with the standard deviation. Statistical analyses were carried out using IBM SPSS Statistics version 27 (SPSS, Inc., Chicago, IL, USA). For comparing the differences among multiple groups, a one-way analysis of variance (ANOVA) was applied, followed by a Tukey’s post hoc multiple comparisons test to determine the specific sources of variation. In cases where only two independent groups were being compared, a two-sample t-test was used. A statistically significant difference was considered when the *p*-value was less than 0.05 (*p* < 0.05).

## 3. Results

### 3.1. Morphological Characterisation of Microcapsules and the Survival Rate under Various Conditions

**Figure 1:**
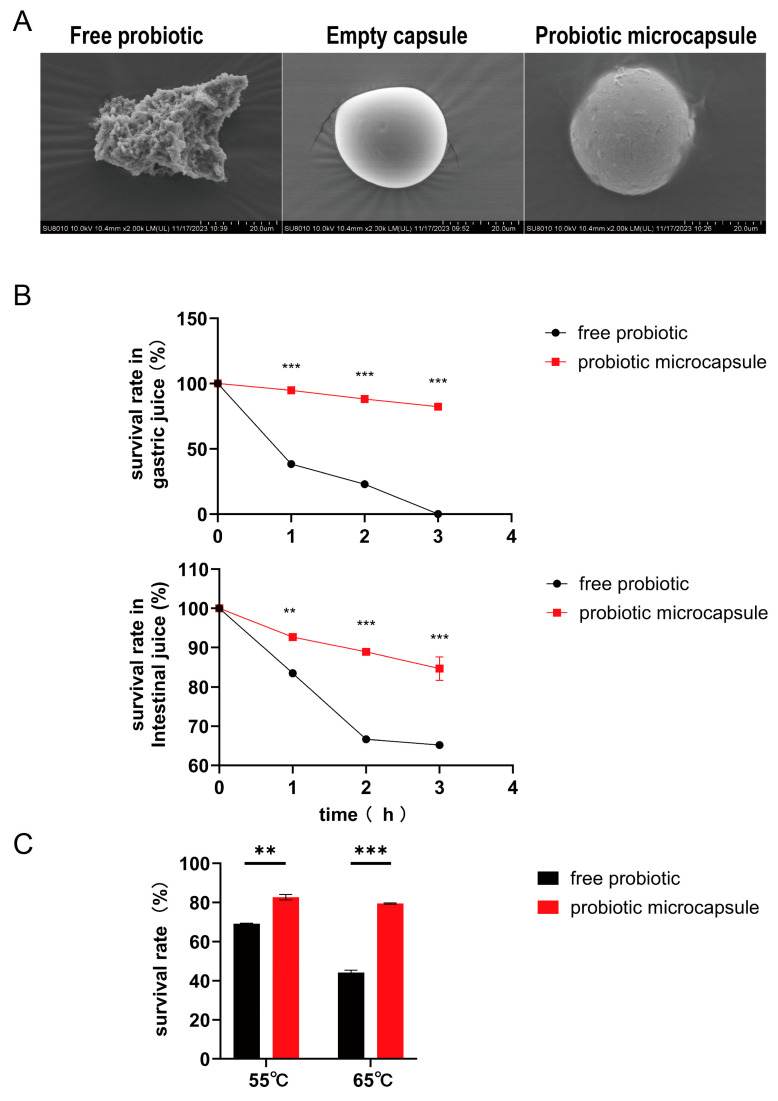
Morphological characterization of microcapsules and the survival rates in different conditions. (**A**) Microstructure of probiotics, empty capsules, and probiotic microcapsules by SEM. (**B**) Survival of probiotic in simulated gastrointestinal conditions. (**C**) Viability of probiotic stored at different temperatures. Data represent at least three independent experiments. (*n* = 3 biological replicates for each group). The data were expressed as mean ± SD. Compared with free probiotic: ** *p* < 0.01; *** *p* < 0.001.

To validate the successful encapsulation process, the lyophilized microcapsules were morphologically characterized using scanning electron microscopy. The resultant data vividly illustrated the presence of a composite-encapsulating layer surrounding the probiotics within the microcapsules (Figure 1A). To assess the viability of microencapsulated probiotic cells in the gastric conditions, P-8 microcapsules were exposed to simulated gastric acids for different periods (1, 2, and 3 h). As presented in Figure 1, the encapsulation technique increased the viability, survival rate, and the tolerance of probiotic cells against gastric conditions. Moreover, they enhanced the viability and thermal stability of probiotic bacteria at high temperatures (as shown in Figure 1C). These findings strongly imply that the microcapsules act as a protective shield to protect the probiotic cells from the aggressive conditions within the gastrointestinal tract, thus significantly boosting their survival rate.

### 3.2. Probiotic Microcapsule Administration Alleviated Colitis

**Figure 2:**
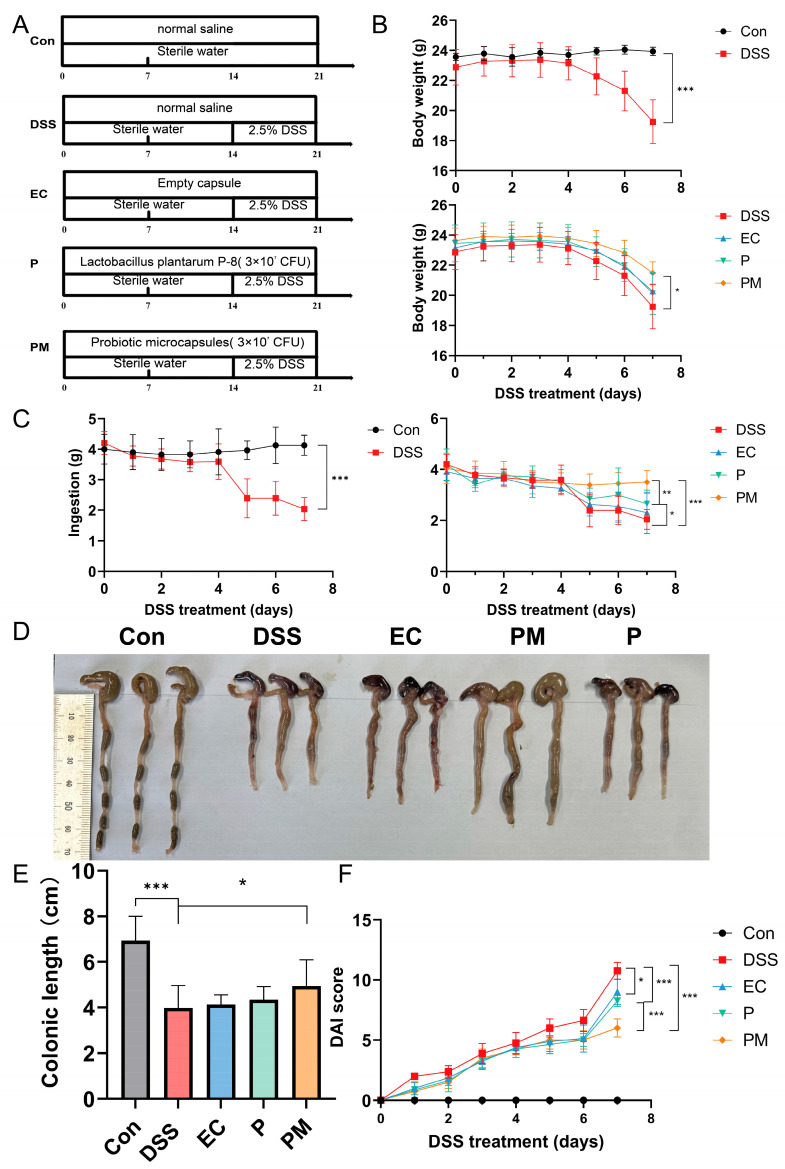
Probiotic microcapsule treatment alleviated dextran sulfate sodium (DSS)-induced colitis in mice. (**A**) Experimental design for supplementing empty capsule, probiotic, and probiotic microcapsule to DSS-induced colitis in mice. (**B**) Mouse body weight. (**C**) Mouse feeding. (**D**) Colon morphology. (**E**) Colonic length. (**F**) DAI scores during DSS treatment stage. *n* = 8 mice/group. The data were expressed as mean ± SD. * *p* < 0.05; ** *p* < 0.01; *** *p* < 0.001.

In our investigation into the therapeutic potential of probiotic microcapsules for treating colitis, we employed oral gavage administration in a murine colitis model. Exposure to dextran sodium sulfate (DSS) resulted in marked decreases in body weight and colon length, concurrent with an increase in the disease activity index (DAI) scores. However, supplementation with probiotic microcapsules notably attenuated these negative impacts (illustrated in Figure 2B–F).

Our findings revealed that the administration of probiotic microcapsules significantly (*p* < 0.05) reduced body weight loss and prevented the shortening of the colon. Furthermore, on the seventh day of DSS exposure, there was a statistically significant difference in the DAI scores between mice receiving P-8 alone (P group) and those given P-8 in microencapsulated form (PM group).

These results collectively indicate that the administration of probiotic microcapsules effectively ameliorated the clinical symptoms associated with UC, suggesting a promising therapeutic role for this targeted delivery method.

### 3.3. Probiotic Microcapsules Alleviated Colonic Mucosal Injury

We subsequently assessed the extent of colonic mucosal damage through histological analysis of the colon. In the DSS and EC group, the histological examination revealed the presence of laminae ulcers, the absence of intestinal glands and epithelium, connective tissue hyperplasia and repair, as well as substantial lymphocyte infiltration. More damages were observed in the submucosa, accompanied by connective tissue hyperplasia and increased lymphocyte infiltration. Additionally, a few dilated intestinal glands with necrotic cell fragments in the glandular cavity were observed. However, the administrations of probiotics and probiotic microcapsules resulted in the significant mitigation of these colonic mucosal injuries, with the probiotic microcapsules being particularly effective in reducing damages.

**Figure 3:**
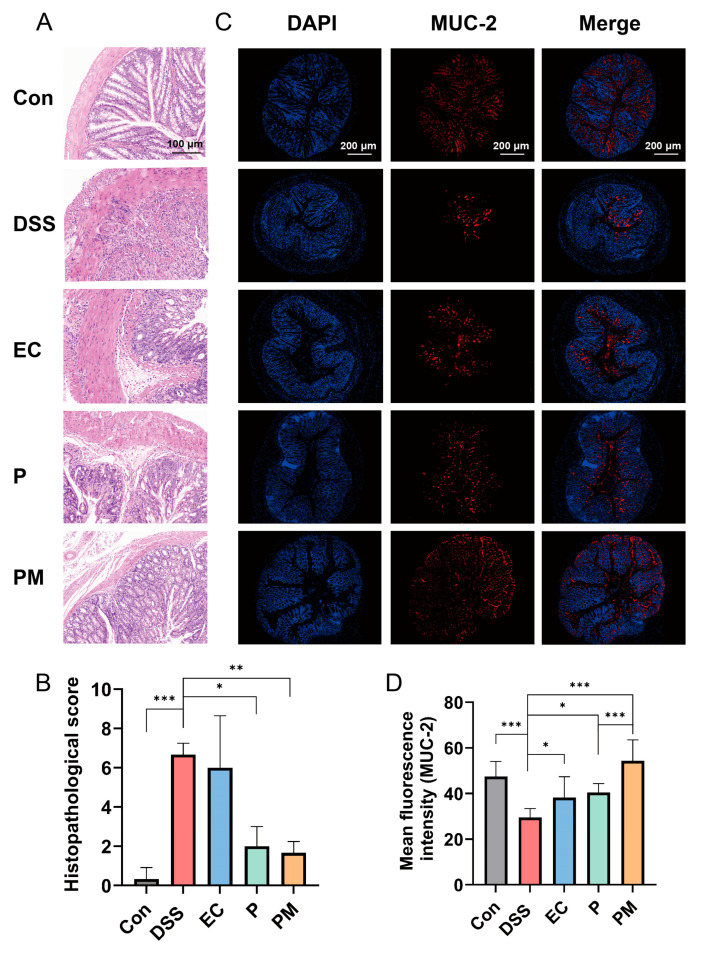
Probiotic microcapsules can alleviate the histopathological lesions of colon and intestinal mucosa. (**A**) Hematoxylin and eosin-stained histological colon tissue, scale bar, 100 μm. (**B**) Histopathological score analysis, *n* = 3 mice/group. (**C**) Immunofluorescence image of colon tissue with MUC-2, scale bar, 200 μm. (**D**) Fluorescence intensity analysis of MUC-2+ mucin, *n* = 3 mice/group. The data were expressed as mean ± SD. * *p* < 0.05; ** *p* < 0.01; *** *p* < 0.001.

Histopathological changes were assessed by the histopathological score of colonic tissue. The histopathological scores in both the probiotic microcapsule-treated mice (1.67 ± 0.58) and probiotic-treated mice (2.00 ± 1.00) were significantly lower than the DSS group (6.67 ± 0.58); there was no significant difference in the EC (6.00 ± 2.65) compared with in the DSS group (Figure 3B).

The effect of probiotic microcapsules on the colon intestinal barrier function was evaluated by detecting MUC-2 expression. MUC-2 expression levels in the PM group were higher than the DSS group, and the fluorescence intensity of MUC-2 in the PM group was significantly higher than that in the P group (Figure 3C,D). These findings suggest that probiotic microcapsules effectively mitigated colonic mucosal injury induced by DSS, and the preventive effect of encapsulated probiotics was better than that of free probiotics. 

### 3.4. Probiotic Microcapsules Alleviated Intestinal Epithelial Barrier Injury by Upregulating TJ-Related Proteins

**Figure 4:**
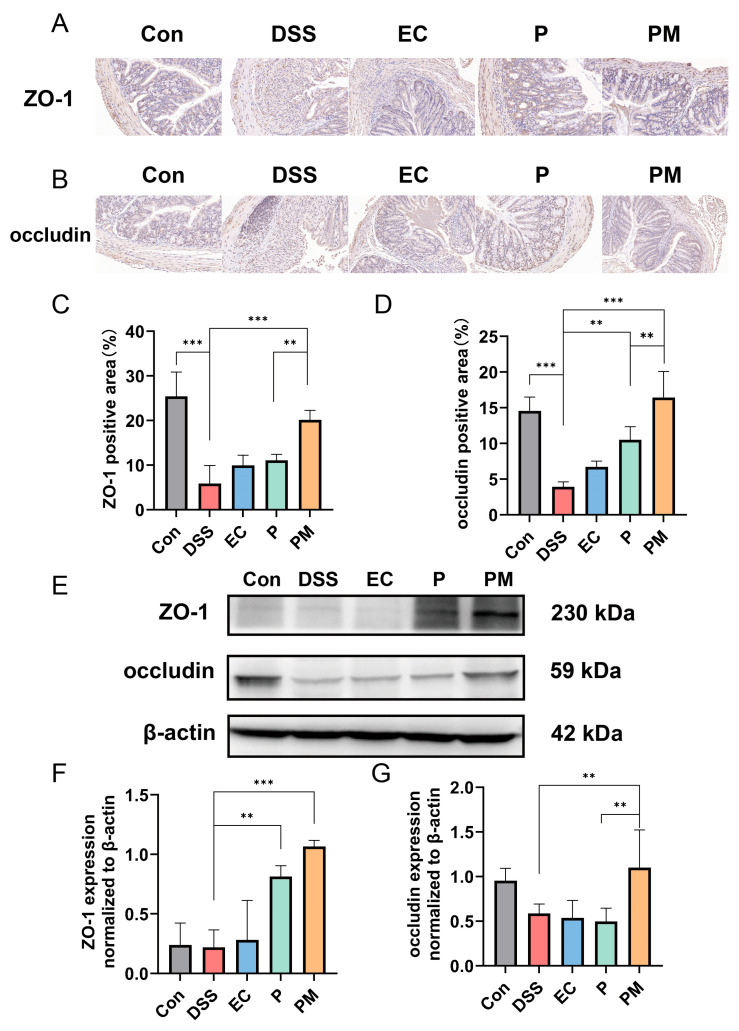
Probiotic microcapsule supplementation alleviated intestinal epithelial barrier damage by regulating the expression of TJ-related proteins. (**A**,**C**), Immunohistochemical analysis of ZO-1, *n* = 3 mice/group (**A**) and occludin, *n* = 3 mice/group (**C**). (**B**,**D**), Positive areas of ZO-1 (**B**) and occludin (**D**). (**E**) Representative Western blots of occludin. (**F**,**G**) Quantifications of the ZO-1, *n* = 3 mice/group (**F**) and occludin, *n* = 3 mice/group. (**G**) Band densities in Western blots. Data represent at least three independent experiments. The data were expressed as mean ± SD. ** p < 0.01; *** *p* < 0.001.

Immunohistochemical (IHC) analysis of the tight junction (TJ) proteins ZO-1 and occludin was conducted to investigate their expression levels in the colitis mice model. Our findings, illustrated in Figure 4A,B, demonstrate a significant downregulation in ZO-1 and occludin in DSS-induced mice when compared to the normal control group. In contrast, the group treated with probiotic microcapsules (PM) exhibited a substantial upregulation in the expression levels of both ZO-1 and occludin (Figure 4B,D). Furthermore, Western blot analysis was performed to assess the expression levels of occludin and ZO-1 in the colon tissue. Consistent with the IHC results, the DSS group displayed a decreased expression of TJ-related proteins, whereas the PM group showed a marked increase in their expression levels (Figure 4E). These data suggest that probiotic microcapsules effectively alleviate DSS-induced injury to the intestinal epithelial barrier by upregulating the expression of both occludin and ZO-1.

### 3.5. Probiotic Microcapsules Alleviated Inflammation by Regulating the Production of Inflammatory Cytokines and Suppressing the NLRP3 Inflammasome Signaling Pathway

**Figure 5:**
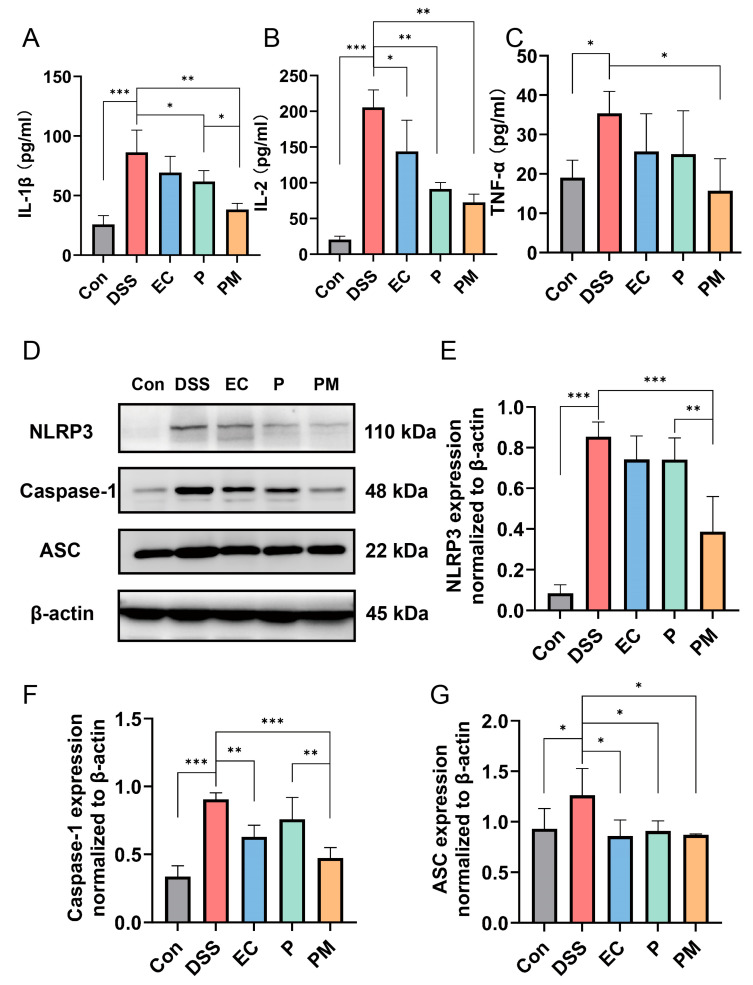
Probiotic microcapsules alleviated inflammation by regulating the production of inflammatory cytokines and suppressing the NLRP3 inflammasome signaling pathway. (**A**–**C**) The levels of IL-1β (**A**), IL-2 (**B**), and TNF-α (**C**) in the serum were measured. (**D**) Representative image of the expression levels of NLRP3, ASC, and caspase-1 detected by Western blotting. (**E**–**G**) Quantification of the NLRP3, *n* = 3 mice/group (**E**), caspase-1, *n* = 3 mice/group (**F**), and ASC, *n* = 3 mice/group (**G**). * *p* < 0.05; ** *p* < 0.01; *** *p* < 0.001.

The effects of probiotic microcapsules on the levels of inflammatory cytokines were investigated. Our data revealed a significant upregulation of IL-1β, IL-2, and TNF-α levels in the colitis mice model. However, the administration of probiotic microcapsules resulted in a notable downregulation in these pro-inflammatory cytokines, as depicted in Figure 5A–C. Interestingly, although groups treated with probiotics also exhibited decreased levels of IL-1β and IL-2, the effect was not as substantial as that seen in the PM group. Moreover, the P group exhibited decreased levels of IL-2 only.

To elucidate the underlying molecular mechanisms of inflammasome inhibition mediated by probiotic microcapsules, we evaluated the activation of the NLRP3 inflammasome signaling pathway through Western blotting. The results indicate a significant elevation in the expression levels of NLRP3, ASC, and caspase-1 proteins in the colon tissues of the DSS group, suggesting an activation of the NLRP3 inflammasome. The administration of probiotic microcapsules effectively inhibited the activation of the NLRP3 inflammasome signaling pathway, which is better than the group treated with probiotics alone (Figure 5D–G). These findings suggest that probiotic microcapsules possess the ability to mitigate inflammation and modulate the NLRP3 signaling pathway.

## 4. Discussion

*Lactobacillus plantarum* P-8 probiotic was isolated and screened from natural fermented sour milk samples of herdsmen families on the grassland of Wulat Zhongqi in Bayannur City, Inner Mongolia [29]. P-8 can inhibit the growth of *E.coli* O157:H7 more effectively than other strains, increase the level of secreted immunoglobulin A in feces of middle-aged and elderly people, and increase the number of bifidobacteria in the intestine [30,31]. The main obstacle to utilizing probiotic bacteria as a food supplement is their sensitivity to harsh conditions such as gastric acids and heat sensitivity. Microencapsulation technology can overcome these challenges by protecting probiotic cells from such aggressive conditions. In this context, many studies have found that encapsulation can improve the viabilities of probiotics when they move through the gastrointestinal tract [32,33]. The gelatin/gum Arabic microcapsules prepared by multiple condensation method have good stability and controlled release performance. Since gelatin and gum Arabic are both natural materials, this method has good sustainability and biocompatibility. In this study, gelatin and gum Arabic were used for probiotic encapsulation, which was optimized on the basis of Min Zhang’s research, and the encapsulation rate of probiotics was as high as 83.9% [25,34]. Then, the physicochemical properties of probiotic microcapsules were studied in vitro. Compared with free cells, the survival rate of cells under simulated gastrointestinal conditions was as high as 82.3% and 84.93% after 3 h, respectively, which could provide effective protection for probiotics. Through the preliminary experiment, 55 °C and 65 °C were selected for a high temperature test, and it was found that the survival rate of probiotic microcapsules at 65 °C was obviously higher than that of unembedded probiotics; the results showed that microcapsule technology effectively protected probiotics against high temperature.

Next, we employed a DSS (dextran sodium sulfate)-induced colitis model to substantiate the colonization capabilities of the probiotic microcapsules in a living organism. The hallmarks of DSS-induced colitis include severe inflammation and damage to the colon lining, such as epithelial erosion and ulceration, the formation of crypt abscesses, the depletion of goblet cells responsible for mucus production, the loss of the protective mucous layer, and the widespread infiltration of neutrophils into the lamina propria—the thin layer of connective tissue beneath the epithelial lining of the intestine [35]. Thus, the histological abnormalities provoked by DSS in mice models of colitis bear a strong resemblance to the clinical presentations typically encountered in human cases of colitis, particularly ulcerative colitis [15]. Therefore, the in vivo results regarding the influence of orally administered *Lactobacillus plantarum* P-8 microcapsules on colonic inflammation indicated that the intake of probiotic microcapsules effectively alleviated several DSS-induced adverse effects, including body weight loss, heightened disease activity index (DAI) scores, shortened colon lengths, and exacerbated histopathological scores reflecting the severity of inflammation. Likewise, the administration of these microcapsules also contributed to the restoration and reinforcement of the intestinal barrier’s structural integrity and increased the levels of MUC-2, ZO-1, and occludin. This implies that the use of *Lactobacillus plantarum* P-8 microcapsules could potentially serve as an effective strategy to alleviate inflammation and maintain gut health in colitis. These results suggest the potential use of probiotic microcapsule to alleviate DSS-induced colitis through the protection of the biological intestinal barrier.

The mucin2 (MUC-2) mucus barrier is the first line of defense that prevents direct contact between colonic epithelial cells and pathogenic microorganism and toxins in the intestines [36]. Several investigations have reported that the development of UC is associated with a decrease in the MUC-2 protein barrier layer in the gut. This decrease leads to severe colonic inflammation with high populations of inflammatory and tolerogenic immune cells [37,38]. In this study, the expression of MUC-2 in the DSS group was significantly lower than that in control group. However, the expression of MUC-2 was significantly increased after administration of probiotic; these results are consistent with previous experimental results, which suggested that probiotics can alleviate mucosal damage in colitis [15,39]. Also, the PM group has a significant difference compared to the P group; these results indicated that probiotic microcapsules had a better protective effect on intestinal mechanical barrier integrity and mucosal barrier function. The intestinal epithelial cells below the mucous layer are connected by TJ-related proteins, such as ZO-1 and occludin, which play a key role in intestinal homeostasis and permeability [6,10]. Therefore, a defective intestinal TJ barrier is an important factor in IBD. The result was as expected, where DSS treatment reduced intestinal barrier function in mice. Probiotic microcapsules can maintain the intestinal barrier function by increasing the expression of ZO-1 and occluding, which may prevent some harmful substances from entering the human body.

Inflammatory responses tend to induce the production of pro-inflammatory cytokines, which promote the development of colitis [15]. Pro-inflammatory cytokines, such as IL-1β, IL-2, and TNF-α, are observably elevated in inflammatory conditions of IBD [15,40,41]; the application of DSS indeed led to a dramatic surge in the expression of IL-1β, IL-2, and TNF-α in the serum samples. Conversely, the administration of probiotic microcapsules was found to significantly suppress the elevation of these pro-inflammatory factors. Compared with both the P group and EC group, the PM group had a more significant effect on inhibiting IL-1β levels. This result confirmed that the encapsulation of probiotics with gelatin and gum Arabic plays a greater role in protecting their viability. Furthermore, IL-1β production through NLRP3 may be involved in the pathogenesis of inflammatory diseases [42,43]. NLRP3 captures danger signals and recruits downstream molecules; inducing IL-1β maturation mediates cytokine release and pyrogenic caspase-1. ASC acts as a bridge between NLRP3 and caspase-1, and these three parts constitute the NLRP3 inflammasome [42,44,45]. Research has evidenced that the suppression or inhibition of NLRP3 activity may significantly decrease the incidence of mucosal barrier dysfunction or damage [46]. To delve into the underlying mechanism by which probiotic microcapsules curb cytokine secretion, we scrutinized their involvement in the NLRP3 signaling pathway. In accordance with the upregulation of IL-1β, the concentrations of NLRP3, caspase-1, and ASC were correspondingly and notably elevated in the DSS-treated group. [15]. Strikingly, probiotic microcapsules can inhibit NLRP3 activation. Moreover, compared with the P group, both NLRP3 and caspase-1 levels in the PM group were significantly decreased. We believe that probiotic microcapsules protect UC by protecting probiotic activity and, thereby, inhibiting the activation of the NLRP3 signaling pathway.

## 5. Conclusions

In conclusion, our research showed that *Lactobacillus plantarum* P-8 microcapsules reduced DSS-induced colitis in mice. Probiotic microcapsule defended the intestinal mechanical barrier integrity and the function of the mucosal barrier. This might be mediated through the downregulation of NLRP3 activation and its signaling pathway. Our results indicated that a probiotic microcapsule is a functional food with great potential to prevent colitis.

## Figures and Tables

**Table 1:** DAI index scoring criteria.

Score	Weight Loss	Stool Consistency	Fecal Occult Blood
0	0	Normal	Normal
1	1–5%	-	-
2	6–10%	Loose stools	Hemoccult positive
3	10–15%	-	-
4	>15%	Diarrhea	Gross bleeding

**Table 2:** Histopathology score system.

Score	Inflammation	Mucosal Damage	Crypt Damage
0	None	None	None
1	Mild	Mucous layer	1/3
2	Moderate	Submucosa	2/3
3	Severe	Muscularis and serosa	100%
4	-	-	100% + epithelium loss

## Data Availability

Data that support the findings of this study are available from the corresponding author upon reasonable request.
